# Hybrid multimodal late fusion frameworks for bvFTD classification in imbalanced dementia datasets

**DOI:** 10.3389/fnagi.2026.1892568

**Published:** 2026-09-16

**Authors:** Majid Ramedani, Jaya C. Terli, Devesh Singh, Oliver Peters, Julian Hellmann-Regen, Josef Priller, Eike Jakob Spruth, Annika Spottke, Anne Boehlen, Patrick Weydt, Ullrich Wüllner, Elisabeth Dinter, Rene Günther, Jens Wiltfang, Björn H. Schott, Emrah Düzel, Wenzel Glanz, Katharina Buerger, Daniel Janowitz, Johannes Levin, Anna Stockbauer, Mihovil Mladinov, Johannes Prudlo, Andreas Hermann, Matthis Synofzik, David Mengel, Gabor C. Petzold, Anja Schneider, Falk Lüsebrink, Stefan Hetzer, Peter Dechent, Michael Ewers, Klaus Scheffler, Sophia Stöcklein, Stefan Teipel, Martin Dyrba

**Affiliations:** 1 German Center for Neurodegenerative Diseases (DZNE), Rostock, Germany; 2 Institute and Policlinic of Radiology, Pediatric Radiology and Neuroradiology, University Medical Center Rostock, Rostock, Germany; 3 Department of Psychiatry and Neurosciences, Charité Universitätsmedizin Berlin, Berlin, Germany; 4 ECRC Experimental and Clinical Research Center, Charité Universitätsmedizin Berlin, Berlin, Germany; 5 German Center for Neurodegenerative Diseases (DZNE), Berlin, Germany; 6 Neuropsychiatry and Laboratory of Molecular Psychiatry, Department of Psychiatry and Psychotherapy, Charité - Universitätsmedizin Berlin, Berlin, Germany; 7 Department of Psychiatry and Psychotherapy, School of Medicine and Health, Technical University of Munich, and German Center for Mental Health (DZPG), Munich, Germany; 8 University of Edinburgh and UK DRI, Edinburgh, United Kingdom; 9 Department of Parkinson's, Sleep and Movement Disorders, Centre for Neurology, University Hospital Bonn, Bonn, Germany; 10 German Center for Neurodegenerative Diseases (DZNE), Bonn, Germany; 11 Department of Neurology, University Hospital Carl Gustav Carus, Technische Universität Dresden, Dresden, Germany; 12 German Center for Neurodegenerative Diseases (DZNE), Dresden, Germany; 13 Department of Psychiatry and Psychotherapy, University Medical Center Goettingen, University of Goettingen, Goettingen, Germany; 14 German Center for Neurodegenerative Diseases (DZNE), Goettingen, Germany; 15 Neurosciences and Signaling Group, Department of Medical Sciences, Institute of Biomedicine (iBiMED), University of Aveiro, Aveiro, Portugal; 16 Leibniz Institute for Neurobiology, Magdeburg, Germany; 17 German Center for Neurodegenerative Diseases (DZNE), Magdeburg, Germany; 18 Institute of Cognitive Neurology and Dementia Research (IKND), Otto-von-Guericke University, Magdeburg, Germany; 19 Institute of Cognitive Neuroscience, University College London, London, United Kingdom; 20 Medical Faculty, Clinic for Neurology, University Hospital Magdeburg, Magdeburg, Germany; 21 German Center for Neurodegenerative Diseases (DZNE), Munich, Germany; 22 Institute for Stroke and Dementia Research (ISD), University Hospital, LMU Munich, Munich, Germany; 23 Department of Neurology, University Hospital of Munich, Ludwig-Maximilians-Universität (LMU) Munich, Munich, Germany; 24 Munich Cluster for Systems Neurology (SyNergy) Munich, Munich, Germany; 25 Department of Psychosomatic Medicine, Rostock University Medical Center, Rostock, Germany; 26 Department of Neurology, University Medical Centre, Rostock, Germany; 27 Department of Neurology, Translational Neurodegeneration Section “Albrecht Kossel”, University Medical Centre, Rostock, Germany; 28 Division of Translational Genomics of Neurodegenerative Diseases, Hertie Institute for Clinical Brain Research and Center of Neurology, University of Tübingen, Tuebingen, Germany; 29 German Center for Neurodegenerative Diseases (DZNE), Tuebingen, Germany; 30 Department of Vascular Neurology, University Hospital Bonn, Bonn, Germany; 31 Department of Old Age Psychiatry and Cognitive Disorders, University Hospital Bonn, University of Bonn, Bonn, Germany; 32 Berlin Center for Advanced Neuroimaging, Charité - Universitätsmedizin Berlin, Berlin, Germany; 33 Department of Cognitive Neurology, MR-Research in Neurosciences, University Medical Center Goettingen, Göttingen, Germany; 34 Department for Biomedical Magnetic Resonance, University of Tübingen, Tuebingen, Germany; 35 Department of Radiology, University Hospital of Munich, Ludwig-Maximilians-Universität (LMU) Munich, Munich, Germany

**Keywords:** 3D-CNN, bvFTD, classification, late fusion, magnetic resonance imaging

## Abstract

**Background:**

Behavioral variant frontotemporal dementia (bvFTD) is an irreversible neurodegenerative disorder characterized by progressive changes in personality and behavior. Magnetic Resonance Imaging (MRI) is widely used to detect and assess structural brain alterations associated with the disease. However, due to the low prevalence of bvFTD among neurodegenerative diseases causing the dementia syndrome, conventional machine learning approaches may struggle to capture comprehensive feature representations. Therefore, this study proposes two late fusion frameworks that integrate a 3D convolutional neural network and a multilayer perceptron (MLP) for improved bvFTD diagnosis.

**Methods:**

A total of 5,928 participants were included, comprising 3,415 healthy controls (HC), 2,276 Alzheimer's disease (AD), and 237 bvFTD, resulting in a class imbalanced setting with bvFTD as the minority class. To address class imbalance, bvFTD data were initially augmented. A 3D-DenseNet was used to extract features from 3D T1-weighted MRI scans, while an MLP-based model was applied to regional brain volumetric measurements obtained from automated MRI-based brain segmentation. Twelve CNN models with different hyperparameter configurations were trained. Models with and without data augmentation, as well as two fusion-based approaches, were evaluated using accuracy, F1-score, and area under the curve (AUC).

**Results:**

Both fusion strategies improved accuracy, F1-score, and AUC compared to the baseline model without data augmentation. Notable improvement was also observed for the bvFTD class, with up to a 120% increase in F1-score. In one of the fusion frameworks, an accuracy of 0.95 ± 0.01 was achieved for bvFTD vs. HC classification. The results demonstrate the effectiveness of the fusion-based approaches compared to non-fused models, outperforming several state-of-the-art methods.

**Conclusion:**

The proposed frameworks demonstrate that data augmentation and fusion strategies can improve accuracy, F1-score, and AUC, with statistically significant gains. Overall, the frameworks improve diagnostic performance and support the identification of relevant biomarkers associated with bvFTD pathology.

## 1 Introduction

Dementia is a clinical syndrome characterized by a progressive cognitive decline, including memory and cognition, that drastically impairs daily functioning and independence (Warren et al., 2024; Halder et al., 2025). Among the various types of dementia, frontotemporal dementia (FTD) is a common cause of early-onset dementia after Alzheimer's disease (AD), with symptoms typically emerging between 45 and 65 years of age (Day et al., 2022). The most prevalent clinical subtype within the FTD spectrum is behavioral variant frontotemporal dementia (bvFTD), which is characterized by progressive behavioral changes and personality disturbances, caused by neurodegeneration in the frontal and anterior temporal lobes (Antonioni et al., 2023). Magnetic Resonance Imaging (MRI) is a non-invasive imaging technique that allows a detailed assessment of brain structure and function by generating high-resolution images, making it a valuable tool in the characterization and diagnosis of neurodegenerative disorders (Huang and Zhang, 2023; Sadu et al., 2025).

Analyzing and diagnosing brain diseases, particularly dementia subtypes, is often time-consuming, complex, and prone to variability and error, as it relies heavily on clinical expertise, and subjective assessment (Wei et al., 2023; Martensen et al., 2026). In recent years, artificial intelligence (AI) has been developed to address these challenges by enabling automated, data-driven analysis of complex medical data (Ahmadi and RabieNezhad Ganji, 2023). More recently, deep learning, particularly convolutional neural networks (CNNs), a class of artificial neural networks within machine learning, has emerged as a powerful approach for medical image analysis (Kshatri and Singh, 2023; Ramedani et al., 2025). CNNs are capable of automatically learning and extracting hierarchical features from imaging data and performing classification tasks, thereby improving efficiency and supporting faster and more accurate clinical decision-making (Teng et al., 2024). This ability to capture complex spatial patterns has made CNNs particularly valuable in dementia research and diagnosis, where subtle structural and functional brain changes must be detected and interpreted. In contrast, multilayer perceptrons (MLPs), another class of deep learning models, are fully connected neural networks that process input data in a flattened form and learn relationships through dense layers (Boccato et al., 2024; Montesinos López et al., 2022). While CNNs are specifically designed to exploit spatial hierarchies in images, MLPs are more suited for structured or tabular data. Combining CNNs and MLPs can therefore leverage both spatial features from imaging data and complementary information from non-image data, leading to improved model performance in complex tasks (Zhang et al., 2026).

Despite recent advances, the classification of dementia subtypes, particularly bvFTD, remains challenging due to several factors. Existing approaches predominantly rely on features extracted at a single spatial scale (e.g., focusing only on regional brain volumes), which may fail to capture complementary information distributed across the brain's hierarchical structure. Therefore, integrating features across multiple views is essential to achieve a more comprehensive characterization of the disease and to improve the accuracy of the model's predictions (Qiao et al., 2026). Hazarika et al. (2023) proposed a hybrid technique incorporating LeNet and AlexNet for AD classification. Waldo-Benítez et al. (2024) investigated the use of deep learning methods, particularly CNN, to classify AD from brain MRI data (Gryshchuk et al., 2025). They compared different approaches and demonstrated that CNN-based models can achieve strong performance. Many existing approaches still rely on limited feature representations or employ 2D CNN architectures, which may not fully exploit the three-dimensional nature of MRI data (Zhang et al., 2024).

Furthermore, due to its relatively low prevalence (accounting for approximately 2% to 5% of all dementia cases), bvFTD is frequently underrepresented in datasets, resulting in a class imbalance problem when training deep learning models (Gonzalez-Gomez et al., 2023; Dattola et al., 2025). This imbalance can bias models toward the majority class, thereby limiting the reliable identification of bvFTD cases (Albattah and Khan, 2025). Di Benedetto et al. (2022) developed a deep learning framework to identify bvFTD. While their dataset was collected from multiple sites, it exhibited only a minor class imbalance. Despite this imbalance, the proposed method achieved acceptable and reliable classification performance. A multimodal computational approach was also used by Donnelly-Kehoe et al. (2019) to identify patients with bvFTD by analyzing sMRI and resting-state functional connectivity. The class imbalance was relatively mild, which allowed the model to achieve good performance. However, in more severely imbalanced scenarios, conventional single-model approaches tend to learn biased decision boundaries favoring the majority class (Chen et al., 2024).

Although multimodal approaches have shown promise in dementia classification, several limitations remain. First, many existing multimodal frameworks combine different imaging modalities or integrate manually extracted biomarkers, while fewer studies have investigated the complementary value of automatically learned spatial representations and anatomically interpretable regional measurements derived from the same MRI modality (Wu et al., 2025; Salman et al., 2025). Second, existing fusion approaches often evaluate a single fusion strategy, leaving the impact of different integration mechanisms on classification performance insufficiently explored (Kahol and Bhatnagar, 2024). Third, the severe scarcity and imbalance of bvFTD datasets remain major challenges, limiting the ability of deep learning models to learn reliable representations for this underrepresented dementia subtype (Beydoun et al., 2026).

To address these limitations, we propose a multimodal MRI-based framework that differs from previous studies in three important aspects. Unlike approaches that primarily combine different imaging modalities or manually engineered biomarkers, our framework integrates two complementary representations extracted from the same structural MRI: automatically learned spatial features generated by a 3D CNN and anatomically interpretable region-of-interest (ROI)-level volumetric features processed by an MLP-based model. While the 3D CNN can learn complex spatial patterns directly from the MRI data, convolutional representations may not explicitly encode anatomically specific information, such as the volume of clinically relevant structures or the degree of regional atrophy. ROI-level volumetric features complement these learned representations by providing explicit, anatomically informed measurements of regional brain structure. This complementary information may help the model capture disease-related atrophy patterns that are not fully represented by the learned spatial features alone. Furthermore, rather than relying on a single feature integration scheme, we systematically investigate two late-fusion strategies to assess how different fusion mechanisms influence classification performance. Finally, to improve the identification of the underrepresented bvFTD class, the proposed framework incorporates targeted data augmentation to mitigate severe class imbalance. We evaluate both the individual and combined contributions of these complementary feature representations for distinguishing bvFTD, AD, and healthy controls (HC).

The main contributions of this study are summarized as follows:(1) We develop a multimodal MRI-based framework that integrates 3D CNN-derived imaging features and ROI-level volumetric features processed by an MLP-based model to improve classification of bvFTD, AD, and HC.(2) We investigate two late-fusion strategies to evaluate different mechanisms for combining heterogeneous MRI representations and assess their effectiveness in improving classification robustness.(3) We address the challenge of severe bvFTD class imbalance through data augmentation and evaluate the proposed framework using a multi-cohort dataset.

## 2 Materials and methods

An overview of the proposed frameworks is shown in Figure 1. The objective of this study is to develop diagnostic frameworks for distinguishing between clinical groups, to evaluate the effectiveness of different late fusion approaches and mitigate the effects of class imbalance in the bvFTD class. We design a 3D CNN-based feature learning model and an MLP-based model to capture complementary information from T1-weighted MRI data at different representational levels. The proposed frameworks consist of three main components. First, a preprocessing step is applied to raw MRI scans to generate gray matter probability maps. Second, a 3D CNN and an MLP-based model are employed to extract multi-view feature representations. Finally, two late fusion approaches are investigated for classification: a Fully-Connected (FC) fusion strategy and an Ensemble-Based (EB) fusion strategy. In the FC approach, a model is trained to perform multi-class classification to simultaneously distinguish between AD, bvFTD, and HC. In contrast, the EB approach evaluates performance on three separate binary classification tasks: AD vs. HC, AD vs. bvFTD, and bvFTD vs. HC.

**Figure 1:**
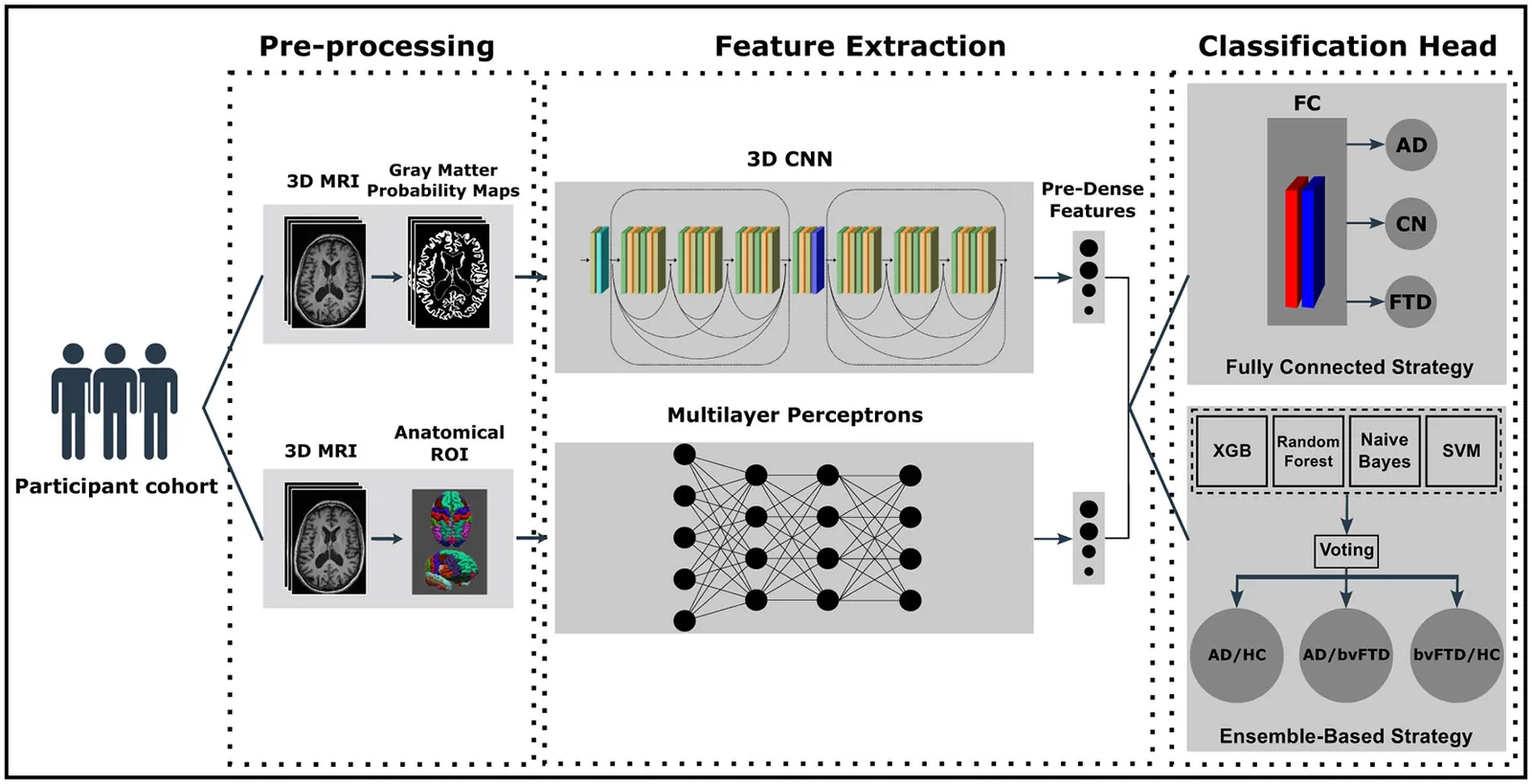
Overview of the proposed multimodal late fusion frameworks for dementia classification. The pipeline consists of three main stages: (I) preprocessing of T1-weighted MRI scans to generate gray matter probability maps, (II) feature extraction using a 3D CNN to learn deep spatial representations and an MLP-based model to process ROI-based volumetric features derived from brain segmentation, and (III) late fusion-based classification. Two fusion strategies are investigated: a Fully-Connected (FC) fusion strategy performing multi-class classification and an Ensemble-Based (EB) fusion strategy evaluated on three binary classification tasks.

### 2.1 Dataset

In this study, we used 3D T1-weighted structural MRI data collected from multiple publicly available databases, enhancing both the generalization ability and classification performance of deep learning models. A key goal was to develop a model that generalizes well to multi-site MRI data for bvFTD identification. To achieve this, we specifically included participants from multiple patient cohorts, each scanned with distinct acquisition devices.

Whole-brain MRI data from 5,928 participants at baseline were used. In total, we included 3,415 HC, 2,276 AD, and 237 bvFTD subjects, drawn from the following sources: Alzheimer's Disease Neuroimaging Initiative (ADNI; http://adni.loni.usc.edu), Australian Imaging, Biomarker & Lifestyle Flagship Study of aging (AIBL; Ellis et al., 2009), Italian Alzheimer's Disease Repository Without Borders (ARWiBo; Frisoni et al., 2009), Latin American Brain Health Institute (BrainLat; Prado et al., 2023), German Center for Neurodegenerative Diseases (DZNE) Clinical Register Study on Neudodegenerative Diseases (DESCRIBE; Hermann et al., 2025), European DTI Study in Dementia (EDSD; Brueggen et al., 2017), Minimal Interval Resonance Imaging in Alzheimer's Disease (MIRIAD; Malone et al., 2013), National Alzheimer's Coordinating Center (NACC; Beekly et al., 2007), Neuroimaging in Frontotemporal Dementia (NIFD; https://memory.ucsf.edu/research-trials/research/allftd), and the Open Access Series of Imaging Studies (OASIS; Marcus et al., 2007).

All imaging data underwent rigorous quality control, and scans with low quality, low signal-to-noise ratio (SNR), or artifacts were excluded. The datasets were then stratified at the subject level and split into training and test subsets to ensure balanced representation of diagnostic classes. Demographic and clinical characteristics of the participants are summarized in Table 1.

**Table 1:** Demographic details of the selected dataset cohorts, distributed over the three classes.

Variables	ADNI	AIBL	ARWiBo	BrainLat	DESCRIBE	EDSD	MIRIAD	NACC	NIFD	OASIS
N (Male)	1535 (779)	606 (263)	806 (272)	385 (159)	119 (66)	498 (247)	63 (29)	1045 (669)	195 (100)	676 (303)
HC/AD/bvFTD	580/955/0	448/158/0	568/210/28	138/164/83	58/0/61	194/304/0	23/40/0	817/226/2	132/0/63	457/219/0
Age (yr)	72.8 ± 7.55	72.8 ± 6.43	52.8 ± 16.62	69.8 ± 9.05	62.2 ± 11.61	70.5 ± 7.33	69.3 ± 7.09	66.5 ± 8.34	62.1 ± 7.06	69.3 ± 8.77

Values are expressed as mean ± standard deviation. N, Number of participants; HC, Healthy Control; AD, Alzheimer's Disease; bvFTD, Behavioral Variant Frontotemporal Dementia.

### 2.2 Data augmentation and pre-processing

As described in Section 2.1, only 237 out of 5,928 samples correspond to bvFTD, i.e., ~4% of the dataset, resulting in a highly imbalanced dataset that may bias the model toward the majority classes. To alleviate this issue, data augmentation was applied exclusively to the bvFTD group in order to increase its effective sample size and improve class balance. Augmentation was performed using the TorchIO library (Pérez-García et al., 2021), where a set of stochastic transformations was defined and applied randomly. Specifically, three augmentation techniques were employed: random elastic deformation (with 8 control points and a maximum displacement of 4.0), random gamma intensity transformation (with log-gamma values in the range [−0.1, 0.1]), and random bias field distortion (with a coefficient of 0.1). For each selected bvFTD sample, one transformation was randomly selected and applied. To avoid excessive augmentation that could lead to overfitting or memorization, augmentation was applied to 140 randomly selected bvFTD samples (approximately 60% of the bvFTD cohort) rather than the entire class. This proportion was selected empirically based on preliminary trials comparing different augmentation levels, rather than through an exhaustive optimization procedure. Following augmentation, 140 additional bvFTD samples were generated, increasing the number of bvFTD samples from 237 (4.0%) to 377 (6.2%) of the total dataset. The selected ratio provided a balance between increasing minority-class representation and preserving a sufficient number of original bvFTD samples. This approach increases variability within the minority class while preserving the original data distribution, thereby improving the robustness and generalization capability of the model. Although augmentation was performed before dataset partitioning, to prevent data leakage, each original image and all of its corresponding augmented versions were treated as a single group during the partitioning process. A group-aware splitting strategy ensured that all members of a group were assigned exclusively to the same data split. Thus, no augmented version of an original image was assigned to a different partition from its corresponding original image or other augmented versions.

MRI scans were preprocessed using an automated in-house pipeline (adapted from Clinica) that integrates tools from the Advanced Normalization Tools (ANTs; Avants et al., 2008) framework and external deep learning-based utilities to standardize images prior to analysis (Figure 2). Gray matter extraction was applied using five consecutive steps. First, bias field inhomogeneity correction was performed using the N4 bias field correction algorithm implemented in the ANTs software package. Second, spatial normalization was implemented by registering each subject's bias-corrected image. Each image was registered to the Montreal Neurological Institute (MNI) standard space using the ICBM152 nonlinear symmetric template (Mazziotta et al., 1995). Third, intensity normalization was applied using ANTs ImageMath, including outlier truncation and global scaling to standardize intensity distributions across subjects. Brain extraction was performed using HD-BET, a deep learning based brain extraction tool. This step removed non-brain tissues and generated subject specific brain masks. Fourth, tissue segmentation was carried out using the ANTs pipeline, which integrates N4 bias correction with the Atropos segmentation algorithm. Segmentation was constrained using the HD-BET derived brain masks and guided by prior probability maps. Brain voxels were classified into three tissue classes: gray matter, white matter, and cerebrospinal fluid. The resulting probabilistic tissue maps, particularly gray matter probability maps, were used for subsequent quantitative analyses. The size of the final probabilistic tissue map is 193 × 229 × 193.

**Figure 2:**
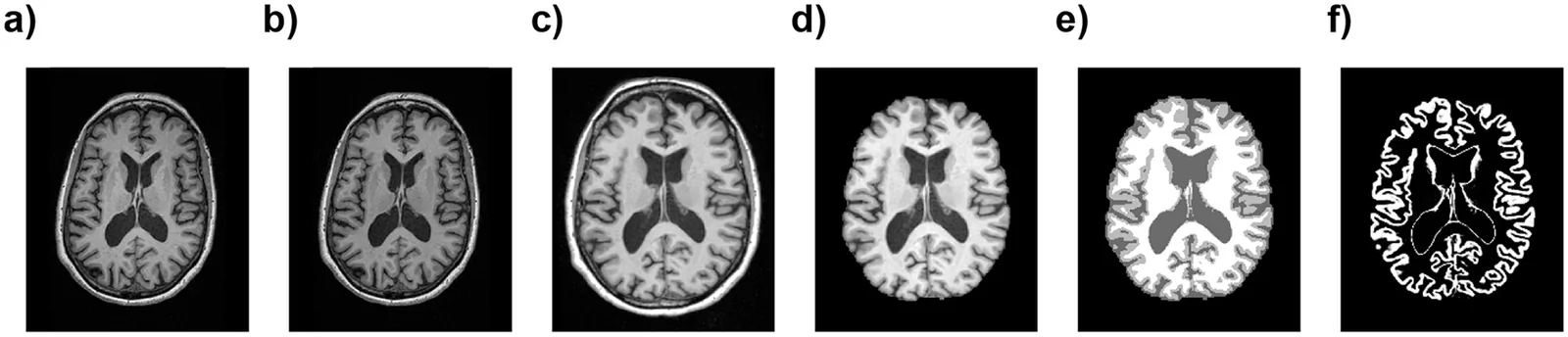
Overview of the preprocessing steps. The figure shows **(a)** the raw data, and the pipeline which includes **(b)** bias field correction, **(c)** registration to the MNI standard space, **(d)** brain extraction, **(e)** tissue segmentation into major brain tissue classes, and **(f)** generation of probability maps for gray matter. For visualization purposes, only representative axial slices from a single subject are shown at each step.

### 2.3 Multi-view feature extraction

#### 2.3.1 Deep 3D CNN-based features

Three-dimensional convolutional neural networks have been widely adopted for dementia classification due to their ability to directly model volumetric brain MRI data (Ganesan et al., 2026; Liang et al., 2021). In contrast to 2D approaches, 3D CNNs can capture spatial dependencies across the full brain volume, enabling more effective learning of distributed disease related patterns. The choice of a three-dimensional Dense Convolutional Network (3D-DenseNet) is motivated by its ability to effectively model complex structural patterns in 3D MRI data (Huang et al., 2016). Unlike conventional convolutional architectures, 3D-DenseNet introduces direct connections between all layers, allowing feature maps to be reused throughout the network (Singh and Dyrba, 2023). This dense connectivity encourages the propagation of both low-level and high-level features. In addition, 3D-DenseNet is known for its parameter efficiency, as it avoids redundant feature learning by reusing previously computed representations. Furthermore, the architecture facilitates improved gradient flow during training, enabling the construction of deeper networks without significant degradation in performance (Zhou et al., 2022).

The proposed 3D-DenseNet model, illustrated in Figure 3, is designed to extract high-dimensional features from T1-weighted MRI scans. The network accepts a single-channel 3D volume as input and begins with a 3D convolutional layer containing 10 filters of size 7 × 7 × 7 with stride 2, followed by a 3D max-pooling layer with kernel size 3 × 3 × 3 and stride 2. The core of the network is composed of multiple dense blocks. Within a dense block, each convolutional layer receives as input the concatenated feature maps from all preceding layers. Each convolutional operation is preceded by batch normalization and a ReLU activation and is followed by a dropout layer (rate = 0.2). The number of layers in each dense block and the number of filters were systematically tuned to balance model complexity and memory efficiency. Between dense blocks, transition layers are applied to reduce the number of feature channels and downsample spatial dimensions. Each transition layer consists of a 3D convolution that halves the number of channels, followed by an average pooling operation with kernel size 2 × 2 × 2 and stride 2. After the final dense block, a global max-pooling operation is applied to aggregate spatial information across the remaining feature maps, producing a fixed-length feature vector that summarizes the learned representations. A ReLU activation and a dropout layer are applied to this vector to further enhance generalization. The flattened feature vector is then passed to a fully connected layer with softmax activation, producing class probabilities.

**Figure 3:**
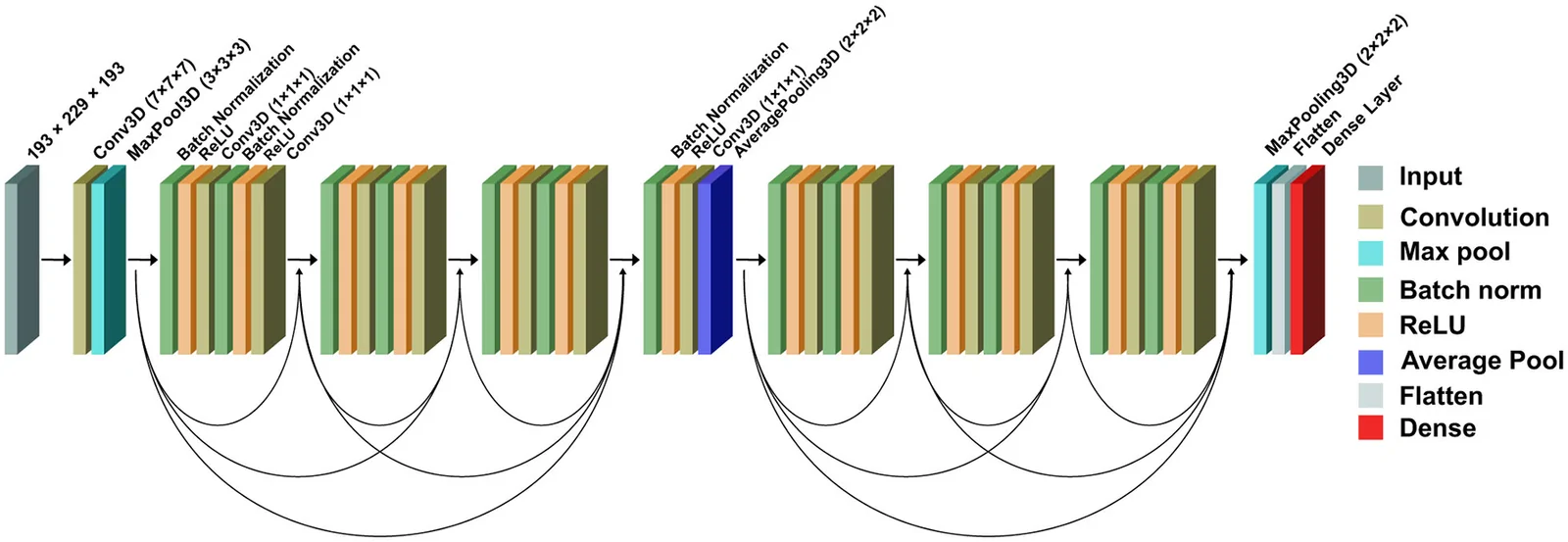
Architecture of the proposed 3D-DenseNet model for deep feature extraction. The network processes 3D MRI data as input and learns discriminative spatial representations through stacked 3D convolutional layers. For clarity, only selected block names and key parameters are displayed above some blocks.

#### 2.3.2 MLP-based features

The MLP-based method was used for volumetric analysis of T1-weighted data. Structural MRI scans were processed using FastSurfer (Henschel et al., 2020) for automated cortical and subcortical segmentation. For each subject, images were parcellated according to the DKT atlas, and volumetric measurements of each region were extracted, including normalization by estimated total intracranial volume (eTIV). The resulting regional volume measurements, together with normalized age and sex, were organized into a structured feature matrix, which served as input to the fully connected MLP. The input to the network consisted of a one-dimensional feature vector for each subject, representing the normalized volumes of cortical and subcortical regions. The MLP architecture comprised four fully connected layers with decreasing dimensionality: 256, 128, 64, and 32 neurons, respectively, each followed by a ReLU activation function. A dropout layer with a rate of 0.3 was applied after the fourth dense layer. The final classification layer consisted of three neurons with a softmax activation function, outputting class probabilities for bvFTD, AD, and HC subjects. A focal loss function (α = 0.1, γ = 2) was employed during training to reduce the influence of class imbalance. The model was trained using the Adam optimizer with the learning rate of 1 × 10^−3^, a batch size of 32, and a maximum of 100 epochs. Early stopping was applied by monitoring the validation loss. The training data were shuffled at each epoch. Feature vectors from the penultimate layer were also extracted for the feature fusion branch.

### 2.4 Classification head (fusion-based strategies)

A fusion pipeline was applied to integrate the complementary information. The extracted features from the 3D CNN and MLP-based models were first converted into fixed-length feature vectors, denoted as *F*_*d*_ and *F*_*m*_, respectively. These modality-specific representations were then concatenated to form a unified feature vector (*F* = *F*_*d*_⊕*F*_*m*_). To ensure balanced integration of heterogeneous feature representations and reduce the influence of differences in feature dimensionality, the modality-specific feature vectors were independently normalized and projected into lower-dimensional embeddings before fusion. Specifically, the CNN-derived and MLP-derived feature vectors were transformed using trainable fully connected projection layers with 128 neurons and ReLU activation. The resulting 128-dimensional representations from both modalities were concatenated to form the fused feature vector, which was then passed to the subsequent classification layers (Li et al., 2025; Chiregi et al., 2025).

#### 2.4.1 Fully connected fusion strategy

Following feature extraction and concatenation, a fully connected neural network was employed to perform multi-class classification of bvFTD, AD, and HC subjects. The fused feature representation integrates complementary information from voxel-wise structural patterns and region-based volumetric measurements.

The concatenated feature vector was first passed through a fully connected layer with 128 neurons and ReLU activation, enabling the model to learn higher-level interactions between modalities. A dropout layer with a rate of 0.5 was subsequently applied to reduce overfitting by randomly deactivating neurons during training. The final classification layer consisted of three neurons with a softmax activation function, producing class probabilities for the three diagnostic categories. The model was trained using the Adam optimizer with a learning rate of 1 × 10^−4^ and categorical cross-entropy loss. To enhance generalization, early stopping was applied based on validation loss, with restoration of the best-performing model weights. Class weights were incorporated during training to mitigate the effects of class imbalance.

#### 2.4.2 Ensemble-based fusion strategy

To further exploit the complementary information in the fused feature space, an ensemble-based classification approach was employed. This approach integrates four classifiers: Support Vector Machine (SVM), Random Forest (RF), Naive Bayes (NB), and eXtreme Gradient Boosting (XGBoost), each contributing distinct modeling capabilities. SVM was selected for its robustness to small sample sizes and its ability to maximize class margins, making it effective in scenarios with overlapping feature distributions (Guido et al., 2024). RF provides robustness to noise and captures feature interactions through ensemble learning (Salman et al., 2024). NB offers a computationally efficient probabilistic framework, which can perform well when feature independence assumptions are approximately satisfied (Schonlau, 2023). XGBoost, a gradient boosting method with regularization, is particularly effective at modeling complex nonlinear relationships and subtle patterns in the data.

The SVM classifier was implemented using an RBF kernel with a regularization parameter of *C* = 1.0, while class balancing was applied to account for imbalanced class distributions. The Random Forest classifier consisted of 300 decision trees with a maximum depth of 20 and class balancing enabled. The XGBoost classifier was configured with 300 estimators, a maximum tree depth of 6, a learning rate of 0.05, and subsampling ratios of 0.8 for both samples and features. The Gaussian Naive Bayes classifier was implemented using its standard formulation without additional hyperparameter tuning.

To combine the predictions from individual classifiers, a weighted soft voting scheme was adopted to account for differences in model performance and reliability. The final prediction is computed as


$$\begin{array}{c} ŷ=\overset{4}{\underset{i=1}{\sum }}w_{i}{ŷ}_{i}, \end{array}$$ (1)

where ŷ_*i*_ denotes the predicted class probabilities from the *i*-th classifier, and *w*_*i*_ represents the corresponding weight. The weights were determined through preliminary pilot experiments comparing several weighting combinations and were fixed before the final evaluation. No further optimization of these weights was performed on the test data. The weights were set to *w*_1_ = 0.3 (SVM), *w*_2_ = 0.2 (NB), *w*_3_ = 0.2 (RF), and *w*_4_ = 0.3 (XGBoost), assigning greater importance to models with stronger capacity to capture nonlinear patterns, while retaining contributions from more interpretable and stable classifiers. These weights were empirically predefined and fixed during evaluation and were not optimized using validation data. The final prediction was obtained by calculating the weighted sum of the predicted class probabilities from the individual classifiers. This ensemble design improves robustness and generalization by leveraging the complementary strengths of diverse learning algorithms.

### 2.5 Network training

All models were implemented using the TensorFlow framework (Abadi et al., 2016). All experiments were performed using fixed random seeds (42) to improve reproducibility. The models were trained with a batch size of 32 to accommodate GPU memory constraints, for a maximum of 100 epochs. Early stopping was applied based on validation loss, with a patience of seven epochs, and the model weights corresponding to the best validation performance were restored. For the 3D CNN branch, regularization techniques were employed to mitigate overfitting. Dropout with a rate of 0.3 was applied after selected convolutional and pooling layers, and the final fully connected layer incorporated combined L1 and L2 regularization (L1 = 1 × 10^−5^, L2 = 1 × 10^−4^) to constrain model complexity. The Adam optimizer was used for training, and categorical cross-entropy served as the loss function. Key hyperparameters were systematically explored, including the number of convolutional filters {8, 12, 16}, the number of DenseNet blocks {3, 4}, and the learning rate {10^−3^, 10^−4^}. In total, 12 distinct model configurations were trained based on the explored hyperparameter combinations. Each of the 12 configurations was independently trained and evaluated using the same predefined dataset partitions. No single model was selected as the final model based on validation performance; instead, the results reported in this study represent the mean ± standard deviation across all evaluated configurations, providing an assessment of model performance variability under different hyperparameter settings.

During the training phase, twelve distinct CNN models, each defined by a different hyperparameter configuration, were trained using the training dataset. The data were partitioned into three non-overlapping subsets: 70% for training, 15% for validation, and 15% for testing. All splits were performed in a stratified manner and kept consistent across experiments to ensure comparability between models. The validation and test sets were strictly separated from the training data and were not used during model optimization. All competing methods were trained and evaluated using identical data partitions to ensure fair comparisons. Performance metrics were computed exclusively on the independent test set.

All experiments were implemented in Python 3.11.14, utilizing TensorFlow 2.20, scikit-learn 1.8, NumPy 2.2.6, xgboost 3.2.0 and aucmedi 0.11.0. The deep learning models were developed using TensorFlow and executed on a Linux server equipped with two Intel Xeon Gold CPUs, 472 GB RAM, and two NVIDIA L40 GPUs. GPU-accelerated training was performed using CUDA-enabled computation.

### 2.6 Interpretability analysis with relevance map

Relevance mapping refers to techniques that determine and visualize which regions of an input image have the greatest impact on a model's prediction (Dyrba et al., 2021). One commonly used approach is Layer-wise Relevance Propagation (LRP), which interprets neural network outputs by propagating relevance scores backward through the model. In this process, the prediction is redistributed from the output layer toward the input, assigning each neuron a score that reflects its contribution to the final decision, which is subsequently mapped onto the input voxels to highlight the most influential regions (Achtibat et al., 2023). In this study, LRP was employed to explain model predictions by iteratively propagating relevance values from the output layer back to the input according to predefined local redistribution rules. The implementation was carried out using the iNNvestigate library (version 2.1.2; Alber et al., 2018). To maintain numerical stability during backpropagation, the LRP-ε rule was applied with a stabilizing term of ε = 1 × 10^−3^. For each input sample, relevance scores associated with a given diagnostic class (AD, bvFTD, or HC) were propagated backward to produce three-dimensional relevance maps, where each voxel represents its contribution to the model's prediction for that class.

### 2.7 Model performance and statistical analysis

Model performance was evaluated using several quantitative metrics derived from the confusion matrix. To obtain a comprehensive assessment of predictive performance, multiple complementary evaluation measures were employed.

- **Accuracy (ACC)** represents the proportion of correctly classified samples among all predictions. While it provides an intuitive overall measure of performance, it can be misleading in the presence of class imbalance.
- **F1-score** defined as the harmonic mean of precision and recall, it provides a balanced measure of classification performance, particularly in datasets with uneven class distributions.
- **Area Under the ROC Curve (AUC)** quantifies the model's ability to distinguish between classes across all possible decision thresholds by evaluating the trade-off between sensitivity and specificity. This metric is independent of any fixed threshold and reflects overall discriminative ability.

Statistical analyses were performed using Python (version 3.11.14), utilizing SciPy version 1.16.3. Results are reported as mean ± standard deviation across the 12 evaluated CNN configurations with different hyperparameter settings. Each CNN configuration was evaluated under three experimental settings: (i) a baseline CNN model trained on the original data without augmentation, (ii) the corresponding CNN model trained with data augmentation, and (iii) the corresponding multimodal fusion model. The same hyperparameter configuration was maintained across the three experimental settings. For model comparisons, the Wilcoxon signed-rank test was used for paired comparisons because the same 12 CNN configurations were evaluated under each corresponding experimental condition. Thus, each statistical comparison consisted of 12 matched pairs (*n* = 12), with each pair representing the performance of the same CNN configuration under the two compared settings. Specifically, ACC, F1-score, and AUC values were compared between the baseline and augmented settings, as well as between the baseline and corresponding multimodal fusion settings. The resulting *p*-values are reported alongside the corresponding comparisons in the Results section. In addition, the 95% confidence intervals for ROC curves and AUC values were estimated using bootstrap resampling. For each bootstrap iteration, test samples were randomly resampled with replacement, and the ROC curve and AUC were recalculated. The confidence intervals were obtained from the 2.5th and 97.5th percentiles of the resulting bootstrap distributions. Statistical significance was defined as *p* < 0.05.

## 3 Results

All twelve models were trained and evaluated within a consistent experimental framework to ensure a fair comparison across different data conditions and fusion strategies. Statistical comparisons between experimental settings were performed using the Wilcoxon signed-rank test, and confidence intervals for ROC curves and AUC values were estimated using bootstrap resampling as described in Section 2.7. Specifically, three experimental settings were considered: (i) a *FC-Baseline* condition, in which CNN models were trained without data augmentation and without any fusion strategy; (ii) an *FC-Augmented* condition, in which models were trained using augmented data but still without applying any fusion strategy; and (iii) a *FC-Fused* setting, in which the augmented CNN models were combined with MLP-derived features using the proposed multimodal fusion approach. The performance of the proposed fusion frameworks was evaluated against progressively enhanced baseline models, including CNN-only models without augmentation, CNN-only models with augmentation, and multimodal fusion models. This experimental design enables assessment of the individual contributions of feature fusion and imbalance mitigation.

### 3.1 Fully connected fusion strategy

To evaluate the effect of data augmentation and multimodal fusion, model performance was systematically compared across the three experimental settings (Table 2 and Figure 4). Statistical significance was assessed using the non-parametric Wilcoxon signed-rank test due to the relatively small sample size and the paired nature of the experiments. All statistical comparisons were performed using the same twelve model configurations to ensure paired evaluation between experimental settings.

**Table 2:** Classification performance across multi-class classification tasks under different experimental settings.

Classes	Setting	F1-Score	AUC	Overall ACC
AD	*FC-Baseline*	0.64 ± 0.031	0.78 ± 0.032	0.71 ± 0.034
bvFTD	*FC-Baseline*	0.39 ± 0.10	0.87 ± 0.02	
HC	*FC-Baseline*	0.78 ± 0.032	0.81 ± 0.02	
AD	*FC-Augmented*	0.62 ± 0.04	0.79 ± 0.02	0.72 ± 0.03
bvFTD	*FC-Augmented*	0.54 ± 0.07	0.90 ± 0.02	
HC	*FC-Augmented*	0.78 ± 0.038	0.83 ± 0.01	
AD	*FC-Fused*	0.67 ± 0.02	0.82 ± 0.01	0.75 ± 0.017
bvFTD	*FC-Fused*	0.66 ± 0.04	0.89 ± 0.01	
HC	*FC-Fused*	0.80 ± 0.02	0.84 ± 0.008	

Values are expressed as mean ± standard deviation. AD, Alzheimer's Disease; bvFTD, Behavioral Variant Frontotemporal Dementia; HC, Healthy Control.

**Figure 4:**
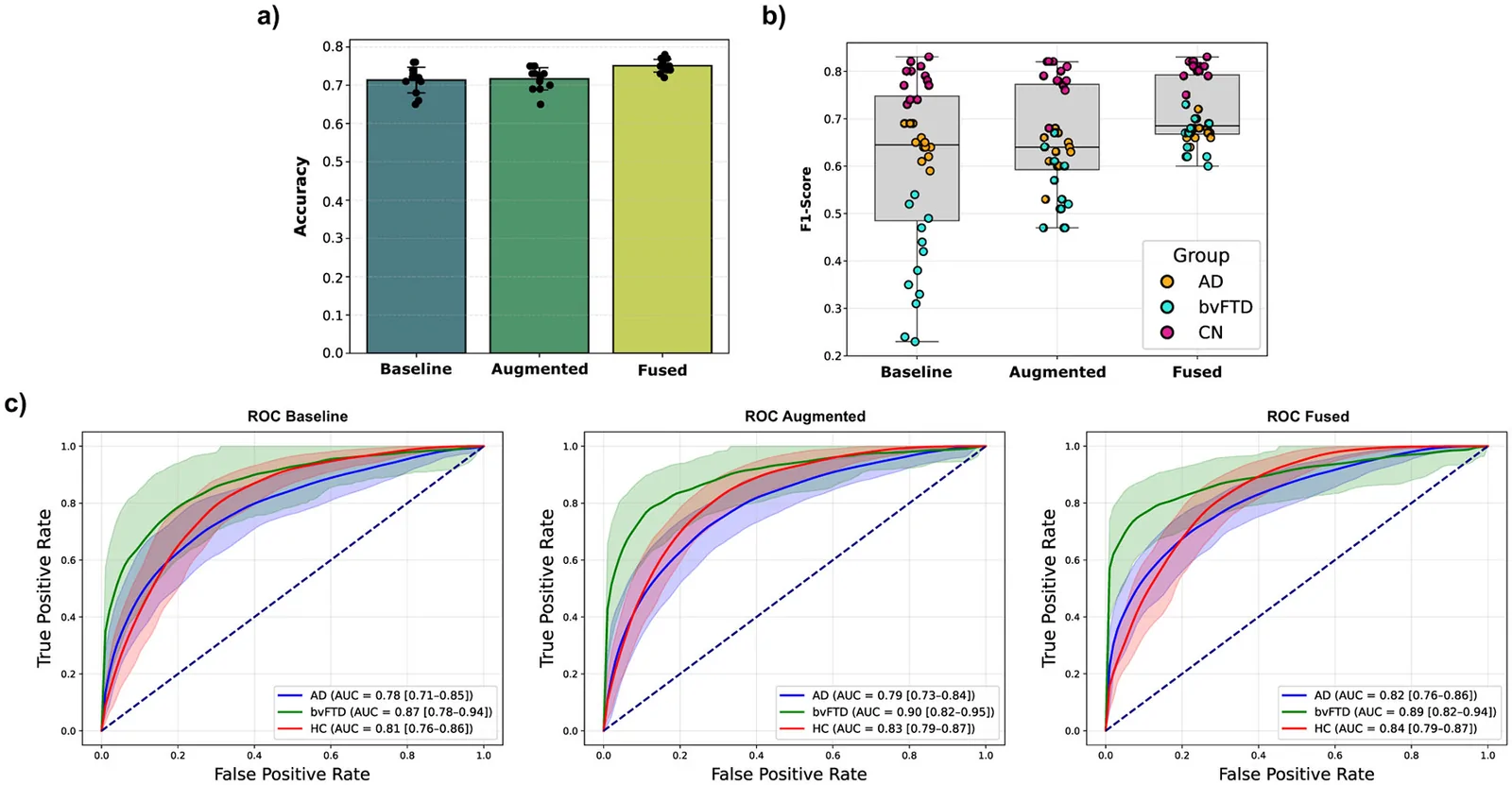
Comparison of model performance across experimental settings: Baseline (no augmentation and no fusion), Augmented (data augmentation without fusion), and Fused (fully connected fusion of CNN and MLP features). **(a)** Overall classification accuracy across the three settings. **(b)** Class-wise F1-scores for AD, bvFTD, and HC across the three experimental conditions. **(c)** ROC curves for each diagnostic class under Baseline, Augmented, and Fused settings, with shaded areas representing the 95% confidence intervals estimated using bootstrap resampling.

The impact of data augmentation was first evaluated by comparing the *FC-Baseline* and *FC-Augmented* CNN models. Overall classification accuracy showed no significant difference between the two conditions (*FC-Baseline* vs. *FC-Augmented*: 0.713 ± 0.034 vs. 0.716 ± 0.030, *p* = 0.79), indicating that augmentation did not substantially affect model accuracy. Compared to the Augmented condition, the Fused model demonstrated consistent performance improvements across all evaluation metrics. Overall accuracy increased significantly to 0.75 ± 0.017 (*p* = 0.006), as illustrated in Figure 4a.

Class-wise F1-score analysis revealed heterogeneous effects across diagnostic groups. No significant changes were observed for AD (*FC-Baseline* vs. *FC-Augmented*: 0.64 ± 0.031 vs. 0.62 ± 0.04, *p* = 0.415) and HC (*FC-Baseline* vs. *FC-Augmented*: 0.781 ± 0.032 vs. 0.784 ± 0.038, *p* = 0.795), whereas a significant improvement was observed for bvFTD (*FC-Baseline* vs. *FC-Augmented*: 0.39 ± 0.10 vs. 0.54 ± 0.07, *p* = 0.001). These results suggest that augmentation primarily benefits the underrepresented or more challenging bvFTD class. *FC-Fused* setting shows significant improvements in comparison to *FC-Baseline* for AD (0.67 ± 0.02, *p* = 0.02) and bvFTD (0.66 ± 0.04, *p* = 0.002), while HC showed no statistically significant change (0.80 ± 0.02, *p* = 0.10). These findings indicate that fusion particularly enhances performance for disease-related classes (Figure 4b).

As illustrated in (Figure 4c) similarly, AUC analysis showed no significant change for AD (*FC-Baseline* vs. *FC-Augmented*: 0.78 ± 0.032 vs. 0.79 ± 0.02, *p* = 0.74), while significant improvements were observed for HC (*FC-Baseline* vs. *FC-Augmented*: 0.81 ± 0.02 vs. 0.83 ± 0.01, *p* = 0.015) and bvFTD (*FC-Baseline* vs. *FC-Augmented*: 0.87 ± 0.02 vs. 0.897 ± 0.02, *p* = 0.013), indicating improved class separability after augmentation. AUC values in *FC-Fused* setting showed a consistent and statistically significant increase across all classes: AD (0.82 ± 0.01, *p* = 0.002), bvFTD (0.89 ± 0.01, *p* = 0.01), and HC (0.84 ± 0.008, *p* = 0.002), confirming improved discriminative capability in the fused model. The shaded regions in the ROC curves shown in Figure 4c represent the 95% bootstrap confidence intervals, providing an estimate of the uncertainty associated with the reported AUC values.

Table 3 summarizes the average relative performance changes across all twelve models. The results confirm that while augmentation yields selective improvements (notably for bvFTD), multimodal fusion provides more consistent gains across all diagnostic categories.

**Table 3:** Average relative performance change compared to the Baseline model across 12 models.

Metric	Setting	Classes		
		AD	bvFTD	HC
F1-Score	*FC-Augmented*	−2.77%	**+52.02**%^**^	+0.36%
AUC	*FC-Augmented*	+1.35%	**+3.20**%^*^	+2.63%^*^
F1-Score	*FC-Fused*	+4.35%^*^	**+79.43**%^**^	+2.93%
AUC	*FC-Fused*	**+6.02**%^**^	+3.10%^*^	+4.64%^**^

Values are reported as a mean percentage changes relative to the Baseline model. AD, Alzheimer's Disease; bvFTD, Behavioral Variant Frontotemporal Dementia; HC, Healthy Control. ^*^P < 0.05, ^**^P < 0.01. The bold values indicate the best performance across the different classification tasks.

### 3.2 Ensemble-based fusion strategy

To further investigate the benefit of combining complementary feature representations, an ensemble-based fusion strategy was applied. This approach was similarly to previous section evaluated under the same three experimental conditions: *EB-Baseline* (no augmentation and no fusion), *EB-Augmented* (data augmentation without fusion), *EB-Fused* (Ensembled-Based fusion of CNN and MLP features).

Table 4 summarizes the classification performance across the three binary classification tasks (AD vs. HC, AD vs. bvFTD, and bvFTD vs. HC) in terms of accuracy (ACC), F1-score, and AUC. Overall, the *EB-Augmented* setting consistently improved performance compared to the *EB-Baseline*, particularly for AD vs. HC and bvFTD vs. HC. These improvements were statistically significant across most metrics (e.g., AD vs. HC: ACC, F1-score, and AUC with *p* < 0.001, *p* < 0.001, and *p* = 0.003, respectively). However, task-dependent effects were observed. While AD vs. bvFTD showed slight variability, bvFTD vs. HC exhibited a pronounced improvement in F1-score, reflecting enhanced sensitivity to the minority class. This suggests that augmentation primarily benefits class imbalance handling and feature generalization.

**Table 4:** Classification performance across binary classification tasks under different experimental settings.

Tasks	Setting	ACC	F1-Score	AUC
AD vs. HC	*EB-Baseline*	0.74 ± 0.01	0.60 ± 0.02	0.80 ± 0.006
AD vs. bvFTD	*EB-Baseline*	0.91 ± 0.005	0.95 ± 0.004	0.85 ± 0.06
bvFTD vs. HC	*EB-Baseline*	0.94 ± 0.013	0.40 ± 0.13	0.90 ± 0.03
AD vs. HC	*EB-Augmented*	0.76 ± 0.01	0.72 ± 0.07	0.82 ± 0.02
AD vs. bvFTD	*EB-Augmented*	0.86 ± 0.06	0.91 ± 0.04	0.86 ± 0.01
bvFTD vs. HC	*EB-Augmented*	0.91 ± 0.06	0.70 ± 0.06	0.92 ± 0.01
AD vs. HC	*EB-Fused*	0.77 ± 0.01	0.70 ± 0.01	0.83 ± 0.006
AD vs. bvFTD	*EB-Fused*	0.90 ± 0.01	0.94 ± 0.01	0.89 ± 0.01
bvFTD vs. HC	*EB-Fused*	0.95 ± 0.01	0.76 ± 0.04	0.91 ± 0.01

Values are expressed as mean ± standard deviation. AD, Alzheimer's Disease; bvFTD, Behavioral Variant Frontotemporal Dementia; HC, Healthy Control.

Importantly, the proposed *EB-Fused* further improved classification performance compared to both *EB-Baseline* and *EB-Augmented* settings. The *EB-Fused* achieved the highest overall performance in most tasks, particularly in AUC and F1-score, indicating improved discriminative power through multimodal feature integration. Minor degradations in ACC and F1-score were observed for AD vs. bvFTD, suggesting that this task remains challenging even with fusion.

To further quantify these effects, Table 5 reports the average relative performance changes across the twelve models with respect to the *EB-Baseline* condition. Overall, augmentation yields substantial gains in F1-score, particularly for bvFTD vs. HC, reflecting improved sensitivity for the most challenging class. In contrast, a slight decrease is observed for AD vs. bvFTD in terms of accuracy and F1-score, indicating that this discrimination remains difficult even after augmentation.

**Table 5:** Average relative performance change in comparison before augmentation across 12 models.

Metric	Setting	Tasks		
		**AD vs. HC**	**AD vs. bvFTD**	**bvFTD vs. HC**
ACC	*EB-Augmented*	**+3.76**%^**^	−5.45%^*^	−3.01%
F1-Score	*EB-Augmented*	+18.53%^***^	−3.40%	**+106.00**%^***^
AUC	*EB-Augmented*	**+2.46**%^**^	+2.20%	+1.27%
ACC	*EB-Fused*	**+4.92**%^**^	−1.05%	+1.09%
F1-Score	*EB-Fused*	+16.60%^**^	−0.87%^*^	**+122.40**%^***^
AUC	*EB-Fused*	+3.47%^**^	**+5.74**%^*^	+1.33%

Values are reported as percentage changes relative to the Baseline model. ACC, Accuracy; AD, Alzheimer's Disease; bvFTD, Behavioral Variant Frontotemporal Dementia; HC, Healthy Control. ^*^P < 0.05, ^**^P < 0.01, ^***^P < 0.001. The bold values indicate the best performance across the different classification tasks.

The *EB-Fused* strategy further enhances performance, especially in AUC and F1-score across all tasks. Notably, bvFTD vs. HC exhibits the largest improvement, confirming that multimodal integration is particularly beneficial for distinguishing clinically similar or imbalanced groups. Overall, these results demonstrate that while augmentation improves class-specific sensitivity, *EB-Fused* provides more consistent and robust gains across all evaluation metrics.

### 3.3 Interpretability of the 3D-DenseNet model

To investigate which brain regions contributed most strongly to the model's classification decisions, relevance maps were generated using LRP, aggregated and visualized for each diagnostic group. Figure 5 presents the mean relevance maps across coronal slices for AD, bvFTD, and HC subjects.

**Figure 5:**
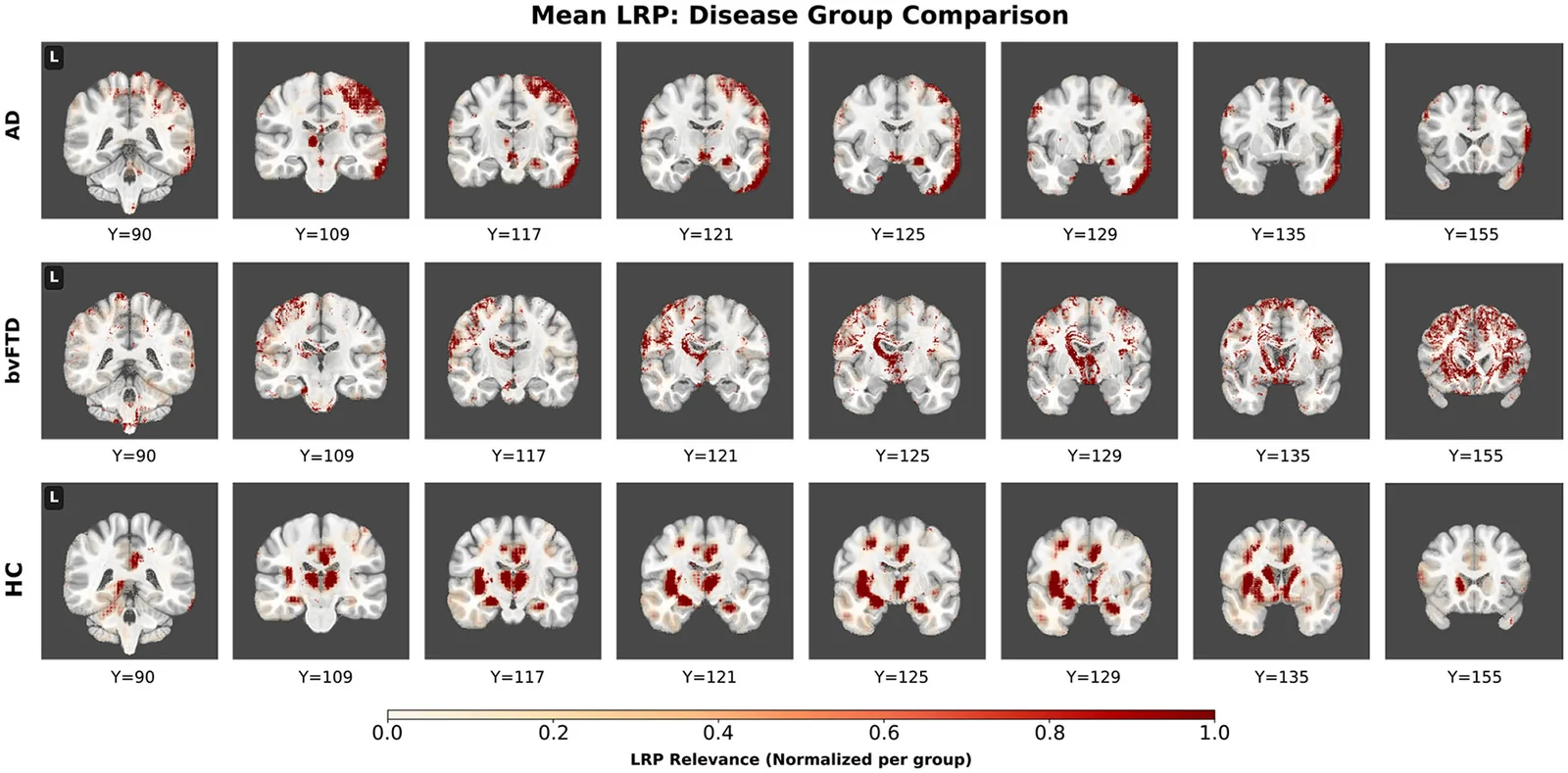
Group-averaged LRP heatmaps across coronal brain slices for Alzheimer's disease (AD), behavioral variant frontotemporal dementia (bvFTD), and healthy control (HC) subjects. Red intensity indicates higher voxel-wise contribution to the model's prediction.

In AD, the model highlighted distributed gray matter regions, including areas within the frontal and parietotemporal lobes, with additional contributions observed in the right hippocampal region. While the relevance patterns are spatially diffuse, they partially overlap with regions known to be affected in Alzheimer's disease (Wu et al., 2021; Gryshchuk et al., 2025). In bvFTD, the most informative regions were concentrated in frontal structures, as well as cholinergic basal forebrain. These regions exhibit measurable microstructural changes during bvFTD. In the HC group, the hippocampus, insular cortex, and thalamus showed high relevance, suggesting that the preservation of these structures contributed to the model's classification performance and its ability to distinguish between healthy and neurodegenerative conditions.

## 4 Discussion

Behavioral variant frontotemporal dementia (bvFTD) is a progressive neurodegenerative disorder primarily affecting the frontal and temporal lobes. Neuroimaging studies consistently report marked atrophy in these regions, often with asymmetric patterns of degeneration. Previous work has predominantly relied on either pathology-driven analyses (Goodwin et al., 2025) or single-view feature extraction approaches (Di Benedetto et al., 2022; Kim et al., 2019), which may not fully capture the complex and heterogeneous nature of bvFTD-related neurodegeneration. In particular, methods based on a single-view may overlook complementary structural and spatial information present in neuroimaging data. To address these limitations, this study introduced two multi-view feature fusion frameworks that integrate complementary representations derived from 3D CNN-based deep features and MLP-based volumetric features. By combining high-level hierarchical imaging features with quantitative structural descriptors, the proposed approach aims to provide a more comprehensive characterization of brain alterations. This integration is particularly relevant in class-imbalanced settings, where bvFTD is underrepresented relative to AD. Such imbalance reflects real-world clinical distributions, in which frontotemporal dementia is less prevalent and may present with overlapping symptoms in certain cases. Robust multi-view feature representations are therefore critical not only for improving classification performance, but also for enabling reliable multi-disease prediction in clinically realistic scenarios. In this context, the proposed approach has broader implications for the development of generalizable models that can operate effectively across heterogeneous and imbalanced neurodegenerative disease cohorts. These findings suggest that integrating complementary MRI representations may have potential as an approach for investigating differential diagnostic classification in settings where disease subtypes exhibit overlapping structural patterns. In particular, the observed performance for underrepresented conditions such as bvFTD indicates a potential direction for further investigation of whether such approaches could contribute to reducing diagnostic bias toward more prevalent dementia types. However, these potential applications require validation in independent, prospective, and clinically representative cohorts before any conclusions regarding clinical utility or effectiveness can be drawn and the result should be therefore interpreted cautiously.

We investigated the diagnostic performance of two late fusion frameworks based on structural MRI for differentiating patients with bvFTD from healthy controls and AD under class-imbalanced conditions. We aggregate ~6, 000 MRI scans from 10 independent datasets, constituting one of the largest multi-site collections used for this task and enabling improved robustness and generalizability across heterogeneous data sources. Twelve CNN models with different hyperparameter configurations were trained on combined dataset, and two strategies were employed to enhance predictive performance, particularly for the minority class. Our results demonstrate that data augmentation, used to synthetically increase the representation of minority samples, leads to improvements in accuracy, F1-score, and AUC. These gains were observed not only in fully connected settings but also in ensemble-based models, where statistically significant improvements were achieved. However, it is important to note that excessive augmentation may introduce unrealistic samples, increase computational complexity, and ultimately impair the model's generalization to real-world clinical data. In both strategies, the observed improvement in the F1-score for bvFTD following data augmentation can be attributed to the enhanced representation of the minority class, resulting in a more balanced decision boundary. The observed improvement following augmentation can be explained by the increased exposure of the model to diverse representations of the minority class, allowing the classifier to learn a more balanced decision boundary. However, because augmented samples are generated from existing observations rather than newly acquired clinical cases, augmentation should be considered a strategy for improving representation learning rather than a substitute for increasing the number of bvFTD samples

Beyond comparisons with previous studies, our experimental design included multiple internal baselines to quantify the contribution of each proposed component. Compared with CNN-only models, multimodal fusion consistently improved classification performance, demonstrating the benefit of integrating spatial deep features with anatomically interpretable volumetric features. Similarly, augmentation primarily improved sensitivity toward the underrepresented bvFTD class, highlighting its role in mitigating class imbalance. In contrast to our study, Meyer et al. used a support vector machine to classify bvFTD vs. controls based on MRI data. Predictions were made at the subject level using whole-brain features, and they achieved an accuracy of 81.7% for distinguishing bvFTD from healthy controls (Meyer et al., 2017). Lampe et al. proposed a multiclass machine learning framework to differentiate seven dementia syndromes using multi-centric structural MRI data. The multi-syndrome classifier achieved an overall accuracy of 47.4%, while the classification performance for bvFTD vs. healthy controls reached 81% (Lampe et al., 2023). Pérez-Millan et al. used cortical thickness and gray-matter volume features with dimensionality reduction and achieved 82.1% accuracy for FTD vs. controls (Pérez-Millan et al., 2023). Gryshchuk et al. employed self-supervised contrastive learning on T1 MRI data across four independent cohorts and achieved a balanced accuracy of 88% for bvFTD vs. controls (Gryshchuk et al., 2025). Goodwin et al. proposed a machine learning framework to differentiate AD and bvFTD using multi-centre neuropsychological and neuropsychiatric data, achieving an average accuracy of 86% based on autopsy-confirmed diagnoses (Goodwin et al., 2025). Singh et al. developed an ontology-based framework using volumetric and cortical-thickness deviation profiles and reported a balanced accuracy of 80% for AD vs. bvFTD classification (Singh et al., 2025). Moguilner et al. (2023) applied DenseNet to raw MRI data and achieved 75% accuracy for bvFTD vs. AD. These studies demonstrate that binary bvFTD classification in our study achieves comparable performance across different methodological approaches. In Table 6, we contextualize the evaluation results of our proposed model with those reported in representative recent studies. The table summarizes the dataset, classification task, MRI modality, feature representation, and performance, thereby highlighting the methodological differences between our approach and previous studies. Because the studies use different datasets and experimental settings, the reported performance values should not be interpreted as direct head-to-head comparisons.

**Table 6:** Comparison of our proposed method with the state-of-the-art.

Study	Cohort	Task	Modality	Features	Performance
Meyer et al. (2017)	52 bvFTD + 52 HC	bvFTD vs. HC	sMRI T1	Voxel-based GM maps	Acc: 81%
Lampe et al. (2023)	426 pts + 51 HC	Binary & multi-class	sMRI T1	Atlas-based volumetry	Bin. Acc: 81% Multi. Acc: 47.4%
Pérez-Millan et al. (2023)	99 HC + 153 AD + 87 FTD	Binary & multi-class	sMRI T1	CTh + subcort. GM volumes	Acc: 63–83%
Gryshchuk et al. (2025)	2,694 (CN, AD, MCI)	Binary & multi-class	sMRI T1-w	SSL ConvNeXt (768-dim)	Bin. Acc: 82–93% Multi. Acc: 78%
Goodwin et al. (2025)	1,498 AD + 118 bvFTD	bvFTD vs. AD	Clinical only	20 neuropsych. features	Acc: 80–90%
Singh et al. (2025)	1,132 MCI + 1,625 HC + 549 AD + 127 bvFTD	AD vs. bvFTD	sMRI T1-w	Vol. + CTh (ontology w-scores)	Acc: 80%
Moguilner et al. (2023)	1,000 bvFTD + 1,000 AD + 1,000 HC	Binary	sMRI T1-w (raw)	3D DenseNet-121 (E2E)	Acc: 92–95%
**Our method**	**3,415 HC** **+** **2,276 AD** **+** **237 bvFTD**	**Binary & multi-class**	**sMRI T1-w**	**Vol**. **+** **3D DenseNet**	**Acc: 71–95%**

AD, Alzheimer's Disease; bvFTD, Behavioral Variant FTD; HC, Healthy Control; MCI, Mild Cognitive Impairment; CN, Cognitively Normal; CTh, Cortical Thickness; GM, Gray Matter; SSL, Self-Supervised Learning; E2E, End-to-End; Vol., Volumetric; Bin., Binary; Multi., Multiclass. The bold values indicate the results obtained using our proposed method.

In this study we employed two late fusion strategies to improve predictive performance by integrating complementary information from MRI data and regional brain volumetric measurements. In the Fully Connected strategy, we evaluated the ability of the models to distinguish among AD, bvFTD, and healthy control subjects. In contrast, an Ensemble-Based framework was further assessed to enable pairwise binary comparisons among these diagnostic groups, thereby providing a more detailed analysis of class separability. Our results demonstrate that, in both strategies, late fusion improves performance and diagnostic accuracy for bvFTD classification under class-imbalanced conditions. Across both fusion strategies, the combination of data augmentation with fusion-based architectures yields superior performance compared to data augmentation alone, highlighting the complementary benefits of these approaches for the classification task. While data augmentation improves the representation of the minority bvFTD class, it may introduce distributional shifts that partially distort class boundaries, particularly between clinically overlapping conditions such as AD and bvFTD, which may explain the observed negative changes in some cases. Additionally, the observed decrease in overall accuracy should be interpreted with caution, as accuracy can overestimate performance in imbalanced settings by favoring majority classes. Although both fusion strategies improve overall performance, direct quantitative comparisons between the FC and EB frameworks should be interpreted with caution due to differences in their classification settings. Despite this limitation, consistent trends can still be observed in the Ensemble-Based approach, which demonstrates more stable improvements across tasks and evaluation metrics, suggesting better generalization. In contrast, the Fully Connected strategy exhibits stronger gains in bvFTD-related F1-score, indicating improved sensitivity to the minority class, albeit with less uniform improvements across all classes.

The interpretability analysis using LRP revealed distinct attribution patterns across diagnostic groups, however, these should be interpreted as model-derived relevance rather than direct neuroanatomical biomarkers. For HC subjects, relevance was primarily concentrated in medial and subcortical regions, including the hippocampus and thalamus, suggesting that the model leverages the preservation of these structures to distinguish healthy controls from pathological groups. In AD, relevance appeared more broadly distributed across cortical regions, particularly in temporoparietal and frontal areas, consistent with the diffuse nature of global brain atrophy (Akl et al., 2025). These patterns may also reflect how the model forms decision boundaries under class-imbalanced conditions. Given the relatively strong and consistent structural signature of AD, the model may implicitly rely on AD-related features, with preserved regions contributing to the differentiation of HC from AD. In contrast, bvFTD relevance maps were more variable and less consistently localized (e.g., Inferior parietal lob, Middle frontal gyrus, Premotor cortex etc.), which may be attributed to the limited sample size, class imbalance, and the known heterogeneity of bvFTD, leading the model to rely on more distributed and less stable features for this class. Although LRP provides useful insight into the model's decision-making process, it should be interpreted as model explanations rather than definitive neuroanatomical evidence.

From a clinical perspective, the proposed framework demonstrates the potential of combining automated imaging representations with interpretable anatomical measurements for supporting dementia subtype classification. Although the model is not intended to replace clinical assessment, such approaches may provide additional quantitative information to assist clinicians, particularly in challenging cases involving overlapping symptoms between AD and bvFTD.

### 4.1 Limitations and future directions

Despite the strengths of our models, several limitations should be acknowledged, which also point to directions for future research. First, although the dataset comprised subjects from multiple cohorts, the overall sample size and cohort diversity remain limited relative to the heterogeneity encountered in real-world clinical populations. In addition, the cohorts were aggregated prior to data partitioning. Consequently, the evaluation represents internal validation on unseen subjects rather than independent external validation on a separate cohort. Differences in demographic characteristics, disease severity, acquisition protocols, scanner characteristics, and clinical populations across institutions may affect model performance and limit generalizability. Future work will assess the proposed frameworks using larger and more diverse independent external datasets from different institutions to further establish their robustness and clinical generalizability. Second, potential selection bias cannot be completely excluded because the study population was derived from the available cohorts and may not fully represent the broader population encountered in routine clinical practice. In particular, differences in recruitment procedures and cohort composition may influence the observed distributions of diagnostic groups and imaging characteristics. Third, the number of bvFTD samples is extremely limited. Although we attempted to mitigate this issue through data augmentation, bvFTD cases still account for only approximately 6% of the dataset, which aligns with the 5-10% of dementia cases being caused by FTD (Olszewska et al., 2016). This substantial class imbalance may bias the model toward the majority classes, particularly HC, rather than enabling it to learn equally discriminative representations across all diagnostic groups. Fourth, the proposed frameworks rely exclusively on structural MRI and ROI-based volumetric measurements. Although these modalities provide complementary structural information, incorporating additional imaging modalities, such as diffusion tensor imaging (DTI), positron emission tomography (PET), or functional MRI (fMRI), could provide additional functional or metabolic information and potentially improve diagnostic performance. Finally, although multiple CNN configurations were evaluated, a broader hyperparameter optimization strategy could identify potentially better-performing architectures. However, exhaustive optimization is computationally expensive, and future work could investigate more efficient optimization strategies, including automated hyperparameter tuning approaches. Importantly, before clinical deployment, prospective multicenter validation will be necessary to establish the clinical utility, robustness, and generalizability of the proposed frameworks. Such studies should evaluate the models prospectively across independent institutions, patient populations, imaging protocols, and scanner platforms, while assessing their performance under real-world clinical conditions.

## 5 Conclusion

In conclusion, this study extends conventional single-modality approaches for bvFTD classification by introducing two multi-view feature learning frameworks. Rather than relying on a single feature type, our findings demonstrate that the complementary integration of multiple features provides a more comprehensive representation of bvFTD-related pathology. This multi-scale fusion improves diagnostic performance for both bvFTD and AD.

## Data Availability

Publicly available datasets were analyzed in this study. This study is based on publicly available datasets, which are available at: Alzheimer's Disease Neuroimaging Initiative (ADNI) (https://adni.loni.usc.edu/data-samples/adni-data), Australian Imaging Biomarkers and Lifestyle flagship study of aging (AIBL) (https://aibl.org.au), Italian Alzheimer's Disease Repository Without Borders (ARWiBo) (https://arwibo.it), Latin American Brain Health Institute (BrainLat) (https://brainlat.uai.cl), European DTI Study in Dementia (EDSD) (https://www.gaaindata.org/partner/EDSD), Minimal Interval Resonance Imaging in Alzheimer's Disease (MIRIAD) (https://www.ucl.ac.uk/brain-sciences/ion/research/research-centres), National Alzheimer'sCoordinating Center (NACC) (https://www.naccdata.org), Neuroimaging in Frontotemporal Dementia (NIFD) (https://memory.ucsf.edu/research-trials/research/allftd), and the Open Access Series of Imaging Studies (OASIS) (https://sites.wustl.edu/oasisbrains).
